# Co-Degradation of Coal and Sawdust for Enhanced Microbial Methane Production

**DOI:** 10.3390/biology14101432

**Published:** 2025-10-17

**Authors:** Liu Zhu, Wangjie Diao, Zeguang Tang, Yi Liu, Yanxin Gu

**Affiliations:** 1 School of Mines, China University of Mining & Technology, Xuzhou 221116, China; zhuliu@cumt.edu.cn (L.Z.); 15929595083@163.com (Z.T.); ts24020156p31@cumt.edu.cn (Y.L.); ts21020106p21@cumt.edu.cn (Y.G.); 2 Yunlong Lake Laboratory, Deep Underground Science and Engineering, Xuzhou 221116, China

**Keywords:** low-rank coal, biomethane, co-digestion, microbial community dynamics, anaerobic fermentation

## Abstract

**Simple Summary:**

It is difficult to efficiently obtain methane from coal using microbes because coal’s hard structure resists breakdown. This study tested whether adding sawdust—a natural waste material—could help microbes break down coal more easily and produce more methane gas. We mixed different amounts of sawdust with coal and measured gas production. We found that adding sawdust greatly increased methane output, especially when the ratio of coal to sawdust in the degraded substrate is 4:1. Sawdust provided easier-to-digest food for the microbes, helping them thrive and work together more effectively. This approach offers a cleaner and more efficient way to turn coal into energy, which could help reduce waste and provide sustainable energy without requiring harsh chemicals or high energy inputs.

**Abstract:**

Microbial coal gasification is a highly promising bioenergy technology, yet its efficiency is often constrained by the highly polymeric structure of coal. This study explores a novel approach to enhance methane production from low-rank coal through anaerobic co-degradation with sawdust. Using Xilinguole lignite as the substrate, we systematically assessed how wood chip supplementation influences microbial degradation efficiency and community dynamics. Results demonstrated that co-degradation significantly increased methane yield—most notably at a coal-to-wood chip ratio of 4:1—far surpassing methane production from coal alone. The addition of sawdust enriched the substrate with bioavailable hydrocarbons and organic acids, and enhanced the degradation of complex compounds including aromatics and lipids. Microbial analysis revealed a marked shift in community structure, with increased abundance of key genera such as *Bacillus*, *Clostridium*, and *Bathyarchaeia*, indicative of enhanced functional specialization and metabolic cooperation. Network analysis further confirmed more tightly interconnected microbial communities in co-degradation systems. These findings underscore the potential of sawdust as a co-substrate to facilitate microbial coal conversion by improving utilization efficiency and promoting synergistic microbial interactions. This strategy offers a practical and efficient means to advance the bioenergy recovery from low-rank coal resources.

## 1. Introduction

The application of biotechnology to achieve clean utilization of fossil fuels has emerged as a highly promising strategy in the field of bioenergy [1]. This strategy aims to depolymerize complex organic macromolecules through the synergistic action of enzyme systems derived from microbial communities. For example, microbial coal gasification relies on multifunctional enzyme systems, such as hydrolases and oxidoreductases, produced by anaerobic microbial communities to convert the organic matter in coal into methane. Compared to traditional physical and chemical gasification methods, microbial gasification operates under mild conditions, is environmentally friendly, and has low energy consumption, thereby offering a promising novel pathway for the clean and efficient utilization of coal resources. However, owing to the highly cross-linked aromatic structures and abundant ether and C–C bonds in coal macromolecules, microorganisms often face issues, such as low substrate accessibility and limited catalytic efficiency. This results in low methane production rates that fail to meet industrial requirements.

Microbial coal gasification is inherently dependent on anaerobic microbial metabolism. The efficiency of this process is highly dependent on the activity of the functional microbial communities. Microorganisms are the core driving unit for the conversion of coal organic matter into gaseous fuels. Moreover, microorganisms are extremely sensitive to environmental conditions and are susceptible to factors, such as pH, temperature, nutrient concentration, redox potential, and the availability of trace elements. Therefore, optimizing and modifying coal properties may improve the efficiency of microbial coal gasification, thereby improving the reaction environment and enhancing functional microorganisms. Suitable strategies include increasing the contact area between coal and microorganisms via mechanical grinding and hydraulic fracturing [2,3], treating coal samples with oxidizing agents or surfactants to increase coal solubility [4,5], applying an electric field to the anaerobic fermentation system to improve electron transfer efficiency [6,7], and pretreating the degradation substrate using bacteria or fungi to enhance coal degradation efficiency [8,9]. Among these strategies, co-degradation technology has improved the stability of anaerobic degradation systems. Additives have a simpler structure than coal. Through anaerobic degradation, they dynamically regulate and stabilize the material exchange process within the reaction system. This reduces the environmental inhibition of microorganisms during the reaction. This inhibition is essentially caused by two factors: environmental conditions deviate from the microbial optimal metabolic range; and harmful substances accumulate that damage cell structures, inhibit enzymes, and interfere with metabolic pathways [9,10,11]. This reduces community function and lowers methane yields.

Lignocellulosic biomass is abundantly available in nature and is an excellent renewable energy source. The biomass is categorized based on origin into agricultural waste (e.g., straw and grain husks), forestry residues (e.g., branches and sawdust), and industrial byproducts (e.g., pulp waste liquid and flax). However, the heterogeneous structure and recalcitrant bonds of lignocellulosic biomass severely hinder its industrial application [12]. As one of the most abundant wastes produced annually in most developing countries, traditional disposal methods for lignocellulosic biomass include uncontrolled dumping, burning, composting, and landfilling [13]. However, all these methods have negative environmental impacts, including greenhouse gas emissions, air pollution, and nutrient loss. Therefore, a rapid, efficient, and sustainable conversion process is crucial for reducing energy consumption and achieving sustainable development of natural resources. Lignocellulosic biomass is pretreated through physical, chemical, and biological pathways [14,15], such as crushing, organic solvents, high-temperature heating, and microbial pretreatment, to render it more amenable to hydrolysis. Furthermore, increasing attention has been paid to the use of hydrogen, methane, and numerous value-added chemicals derived from the depolymerization process [16,17].

Although previous research has shown that co-degrading lignocellulosic biomass like straw with coal can increase methane production [18,19,20], few studies have examined coal biodegradation with sawdust—a forestry waste material rich in lignin and structurally similar to coal. On this basis, fewer have focused on microbial community changes and system stability. Unlike straw, sawdust contains more aromatic polymers and is harder to break down. This likely shifts microbial metabolic pathways and encourages the growth of species specialized in decomposing lignin. It is worth noting that sawdust acts as an excellent carbon source for microorganisms while also stabilizing fermentation pH, which helps maintain microbial activity [21].

This study aims to fill this gap by examining how coal and sawdust together improve methane production under anaerobic conditions. We focus on how sawdust serves as both a carbon source and a buffer, and we optimize key process conditions to increase methane yield. Our work provides new ideas for converting hard-to-degrade carbon sources and supports a sustainable strategy for more efficient coal bioconversion.

## 2. Materials and Methods

### 2.1. Sample Collection and Preparation

The coal sample used in this study was lignite, collected from the Baiyinhua Coal Mine in the Inner Mongolia Autonomous Region, China. The sawdust employed in the experiments was derived from pine wood obtained from a local timber processing plant in Xuzhou, characterized by high contents of cellulose and lignin. After drying, the samples were sieved through a 200 mesh for further tests. Industrial analysis of the coal samples was conducted using a 5E-MAG6700 fully automatic industrial analyzer (Changsha Kaiyuan Instruments, Changsha, China) (Table 1 and Table 2).

### 2.2. Methanogen Enrichment and Cultivation

Culture medium preparation and methanogen enrichment were performed as previously described [22].

### 2.3. Gas Production Evaluation

Saltwater bottles (250 mL) were used as reactors, and Xilinguole lignite was used as the coal sample. To maintain a total fermentation substrate mass of 10 g, coal and sawdust were weighed and added to the fermentation bottles at mass ratios of 4:1 (MM group), 3:1, 2:1, and 1:1 (MT group). Control groups containing pure coal (KB group) and sawdust (MX group) were also established. Thereafter, 50 mL of culture medium was injected into each bottle, and sterilization was carried out at 120 °C for 15 min. After the temperature of the fermentation bottles decreased to 45 °C, 0.5 mL of vitamin solution, 0.25 mL of trace metal element solution, and 50 mL of bacterial inoculum were sequentially added. The bottles were evacuated, sealed with butyl rubber septa, and incubated in a constant-temperature environment at 35 °C.

### 2.4. Instruments and Methods

#### 2.4.1. Gas Analysis of Degradation Systems

Total gas production and gas production efficiency of anaerobic fermentation were measured using an RTK-BMP automatic methane potential test system (Rocktek, Hubei, China). Gas production in the experimental bottles was monitored using a connected channel. The gas components and concentrations were analyzed using a GC9790plus gas chromatograph (Fuli Instruments, Wenling, China). Samples were manually injected at a volume of 1 mL.

#### 2.4.2. Analysis of Key Liquid-Phase Products

Total gas production and gas production efficiency of anaerobic fermentation were measured using an RTK-BMP automatic methane potential test system (RockTek, Wuhan, China). Gas production in the experimental bottles was monitored using a connected channel. The gas components and concentrations were analyzed using a GC9790plus gas chromatograph (Fuli Instruments, Taizhou, China). Samples were manually injected at a volume of 1 mL.

#### 2.4.3. Cellulose Content Quantification

The dried fermentation residue was mixed with phenol-sulfuric acid reagent and heated in a constant-temperature water bath at 80 °C for approximately 30 min. After cooling to room temperature with cold water, 1% aqueous phenol solution was added for colorimetric determination. The cellulose content was calculated based on colorimetric results.

#### 2.4.4. Calorific Value Analysis of the Solid Residue

A unit mass of fermented substrates with different ratios was placed in an oxygen bomb under pressures ranging between 2.9 and 3.1 MPa. The oxygen bomb was immersed in water inside an inner bucket, and the mass of the inner bucket water was kept constant across all the test groups. A sufficient amount of oxygen was introduced to ensure complete combustion of the solid residue. The heat released during combustion was absorbed by the water in the inner bucket, and the calorific value of the solid residue was calculated based on the temperature change in the inner bucket water.

#### 2.4.5. Microbial Community Sequencing

An Illumina high-throughput sequencing platform (Shanghai Meiji Biological, Shanghai, China) was used to analyze the methanogenic bacterial communities before and after polymerase chain reaction (PCR) amplification. The 338F_806R primer sequence was used for bacterial amplification, whereas the 524F_958R primer sequence was used for archaeal amplification. The PCR products were identified via agarose gel electrophoresis. After sequencing, sequence clustering was performed with 97% similarity to obtain operational taxonomic units (OTU), and the most abundant sequence was selected as the representative OTU sequence.

## 3. Results

### 3.1. Gas Production Characterization

**Figure 1:**
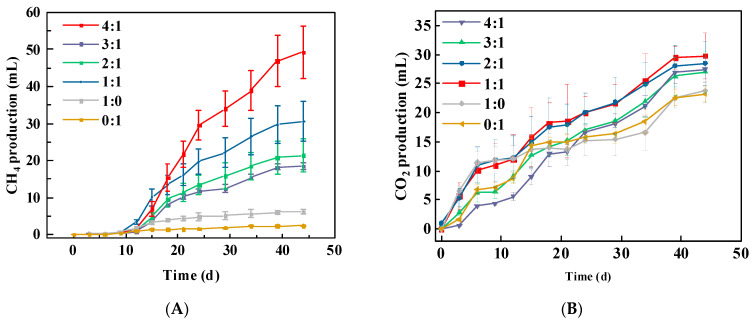
Methane (**A**) and carbon dioxide (**B**) production during the co-degradation of lignite and sawdust.

Addition of sawdust substantially enhanced the efficiency of methane production from coal (Figure 1A). Different mixing ratios resulted in notable differences in the methane yield. The highest methane production, which reached 49.28 mL, was observed at a coal-to-sawdust ratio of 4:1, followed by that at a 1:1 ratio. Methane production exhibited a three-phase pattern: an initial slow increase, a rapid rise, and final stabilization. In contrast, the CO_2_ production in the control groups ranged between 22 and 25 mL, which was slightly lower than that in the experimental groups (23–32 mL) (Figure 1B), with no considerable difference observed. The CO_2_ production demonstrated a steady increasing trend.

### 3.2. Liquid Organic Matter Analysis

**Figure 2:**
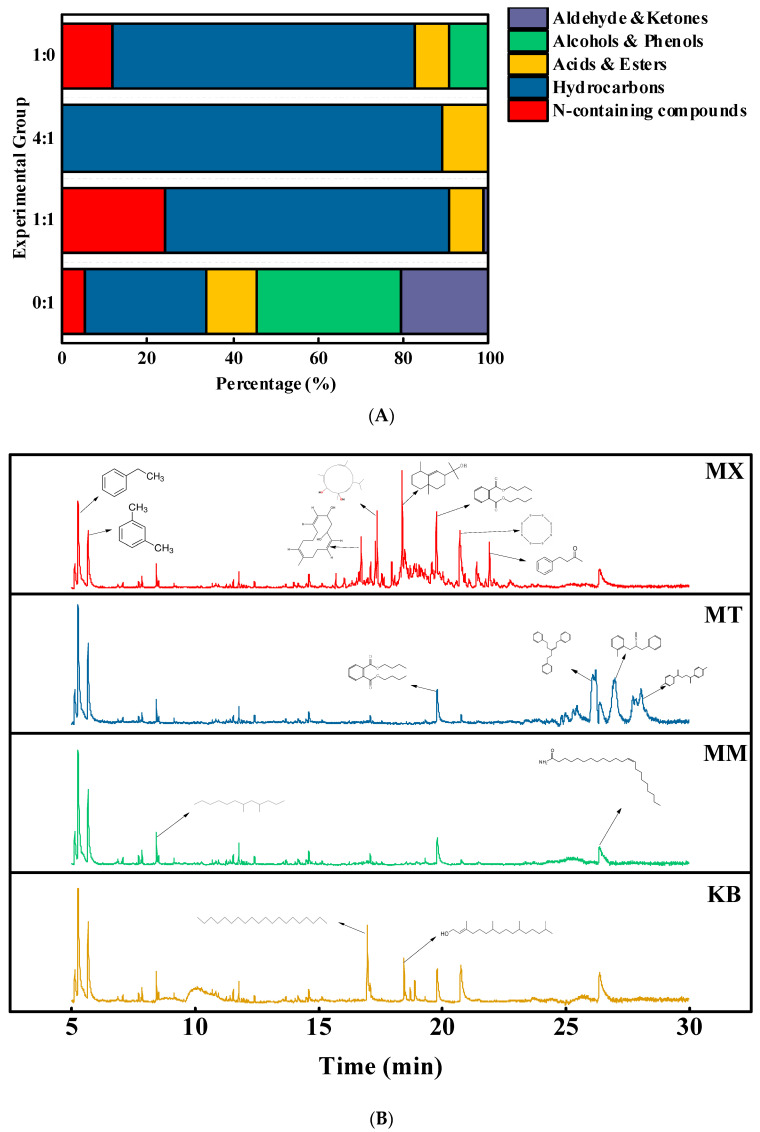
Organic compound composition (**A**) and chromatograms (**B**) in the fermentation broth of different experimental groups as determined via gas chromatography–mass spectrometry (GC-MS) analysis. KB, pure coal group; MM, coal-to-sawdust ratio of 4:1; MT, coal-to-sawdust ratio of 1:1; MX, pure sawdust group.

To highlight the differences in the experimental outcomes, the MM and MT groups were selected as representatives of the mixed methane-producing groups and compared with the control groups. The generation and consumption of organic compounds in batch reactors, also indicate the promoting or inhibiting effects of sawdust on coal biomethanation. The changes in the organic compounds in the solution at the end of the reaction were analyzed using gas chromatography–mass spectrometry. Seventy compounds were detected and categorized into the following five classes: nitrogen-containing compounds, hydrocarbons, acids and esters, alcohols and phenols, and aldehydes and ketones (Figure 2A). The organic compounds with a content greater than 1% are listed in Table 3. Owing to the chemical inertness resulting from high-energy carbon-hydrogen bonds, the mixed groups exhibited fewer types of organic compounds than those of the control groups, with the majority being hydrocarbons. Notably, an increase in the coal-to-sawdust ratio promoted the degradation of nitrogen-containing compounds, ketones, and aldehydes. The aromatic compounds showed a pronounced dose-effect relationship, with their abundance decreasing as the proportion of coal increased, thereby indicating enhanced degradation (Figure 2B). In the MM group, only hydrocarbons, acids, and esters remained in the solution at the end of degradation. To some extent, the chromatographic profile of the mixed groups was closer to that of the pure coal group, but it was simpler than that of the pure coal group. This indicates that during co-fermentation, compared with coal, the organic matter in sawdust (such as cellulose and hemicellulose) is more easily degraded, and the resulting small-molecule compounds serve as a carbon source to sustain biological activity [23,24], while the recalcitrant organic matter in coal, such as aromatic compounds, although it decomposes slowly, remains an important precursor for methane production [25].

### 3.3. Cellulose Content and Calorific Value Analysis

**Figure 3:**
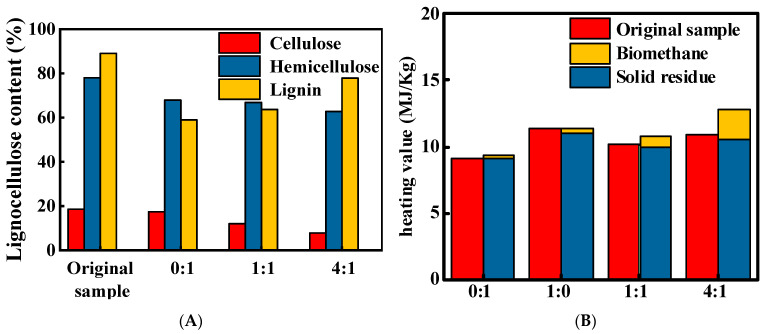
Effect of coal-to-sawdust ratio on (**A**) relative lignocellulose content in solid products after anaerobic degradation and (**B**) solid residue and biomethanation calorific value.

The total lignocellulose content in the solid products decreased relative to the original sawdust, as revealed by quantitative analysis (Figure 3A). Both cellulose and hemicellulose contents showed a negative correlation with the coal-to-sawdust ratio. Specifically, cellulose conversion increased from 6.5% to 58%, whereas hemicellulose conversion increased from 13% to 19.5%. Conversely, the lignin content exhibited a positive correlation with this ratio; as the proportion of coal in the degradation matrix increased, the lignin conversion gradually decreased from 33.8% to 12.5%. Figure 3B compares the calorific values of the solid products and the biomethanate before and after the reaction. The 4:1 experimental group achieved the highest total calorific value of 12.8 MJ/kg with a 17.43% yield increase, whereas the 1:1 group reached 10.74 MJ/kg with a 5.29% yield increase.

### 3.4. Microbial Community Analysis

**Figure 4:**
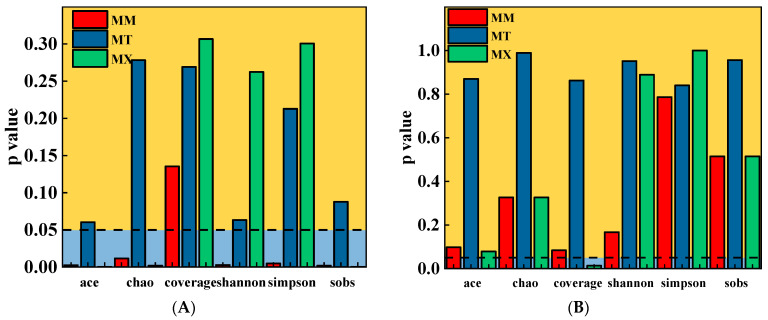
One-way multivariate analysis of variance (MANOVA) of bacterial (**A**) and archaeal (**B**) α-diversity under co-degradation conditions with different coal-to-sawdust ratios. MM, coal-to-sawdust ratio of 4:1; MT, coal-to-sawdust ratio of 1:1; MX, pure sawdust group. The dashed line represents the case where the p-value is equal to 0.05.

Statistical analysis was performed using IBM SPSS Statistics 27 software. The KB group was designated as the control group, while the MT, MM, and MX groups were set as comparison groups. Post hoc multiple comparisons were carried out using Dunnett’s method, with three replicate samples included in each group. In terms of bacteria (Figure 4A), the MM group showed a significant difference in species richness compared with that of the control group (*p* > 0.05, ACE and Chao1 indices), whereas the MT and MX groups showed no significant differences in species diversity (*p* > 0.05, Shannon and Simpson indices). In terms of Archaea (Figure 4B), no significant differences in species diversity or richness were observed between any experimental group and the KB group (*p* > 0.05).

**Figure 5:**
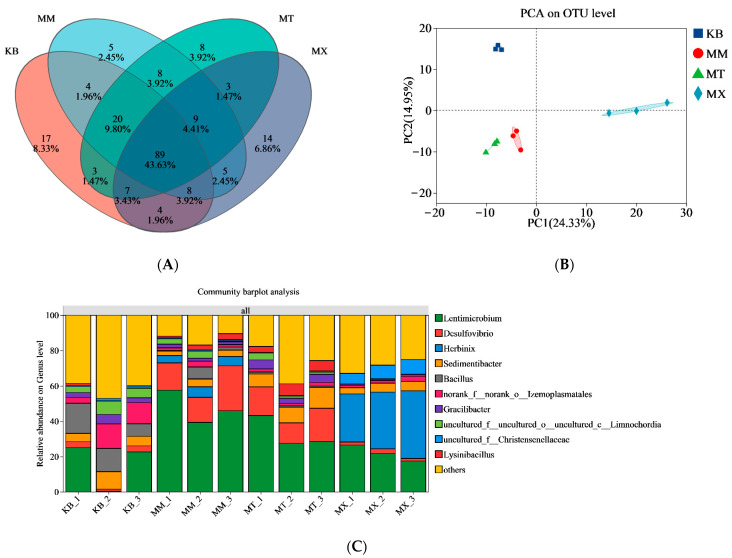
Characteristics of bacterial communities at the genus level in the fermentation broth after co-degradation with different coal-to-sawdust ratios. Species with a relative abundance of less than 1% are classified as “others.” (**A**) Venn diagram, (**B**) Principal component analysis (PCA) plot, the origin (0,0) where the dashed lines intersect in the figure represents the average value of all samples and serves as the reference center for determining the deviation of each sample point from the average level, (**C**) Dominant bacterial genera and their abundance levels.

A total of 205 bacterial genera were identified. Except for the co-fermentation groups, which showed relatively similar compositions, notable differences were observed among the experimental groups (Figure 5A,B). In the KB group, the post-reaction community composition was relatively complex, with minor differences in the proportions of the various genera (Figure 5C). In the MX group, *Lentimicrobium* (17.49–26.60%) and *Herbinix* (27.22–38.30%) were the dominant genera. The relative proportions of the microbial communities changed substantially after co-fermentation of coal and sawdust. In the MT group, *Lentimicrobium* (27.63–43.25%), *Desulfovibrio* (14.25–25.28%), and *Sedimentibacter* (7.21–11.88%) were dominant. Notably, the dominant genera in the MM group were similar to those in the MT group; however, differences in the substrate ratios led to variations in their abundance. Specifically, the abundance of *Lentimicrobium* (39.40–57.64%) increased considerably, whereas that of *Sedimentibacter* (2.60–4.39%) decreased compared with that of the MT group. Additionally, *Herbinix* (4.15–5.90%) was present in the MM group.

**Figure 6:**
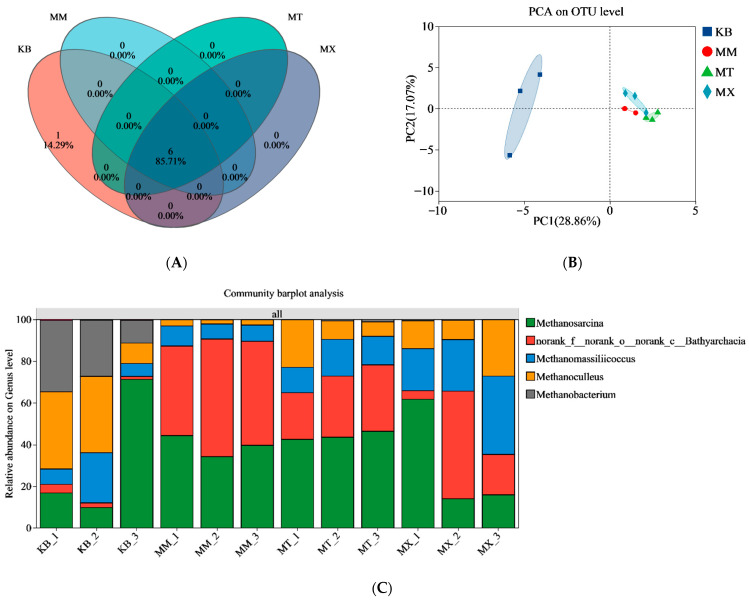
Characteristics of archaeal communities at the genus level in the fermentation broth after co-degradation with different coal-to-sawdust ratios. Species with a relative abundance of less than 1% are classified as “others.” (**A**) Venn diagram, (**B**) Principal component analysis (PCA) plot, the origin (0,0) where the dashed lines intersect in the figure represents the average value of all samples and serves as the reference center for determining the deviation of each sample point from the average level, (**C**) Dominant archaeal genera and their abundance levels. OTU, operational taxonomic unit; KB, pure coal group; MM, coal-to-sawdust ratio of 4:1; MT, coal-to-sawdust ratio of 1:1; MX, pure sawdust group.

Seven archaeal genera were identified. The biological composition was similar among the MM, MT, and MX groups, whereas the KB group was distantly separated from the others, indicating considerable differences in community composition (Figure 6A,B). The dominant archaeal communities in the MM, MT, and MX groups were similar in type but differed in abundance (Figure 6C). In the coal-sawdust co-fermentation groups, the abundance of *Methanosarcina* (34.16–46.38%) was comparable. The MM group exhibited a high abundance of an unclassified genus under Bathyarchaeia (42.88–56.40%), whereas the MX group showed a higher abundance of *Methanomassiliicoccus* (20.14–37.60%). The abundance of *Methanoculleus* (6.88–26.89%) in the MX group was similar to that in the MT group. In contrast, the KB group was characterized by a high proportion of *Methanobacterium* (10.97–34.22%) among the dominant genera, whereas the unclassified genus under *Bathyarchaeia* was scarcely present (1.42–4.12%). The archaeal community distribution of the KB group showed higher similarity with that of the MT group than with that of the MM group.

**Figure 7:**
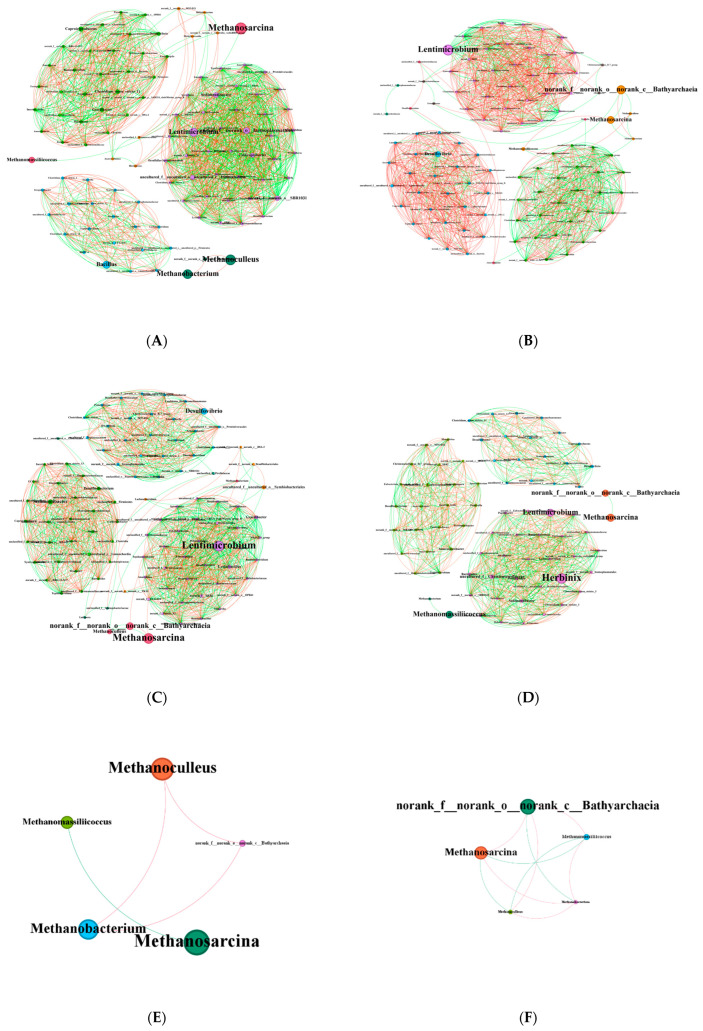

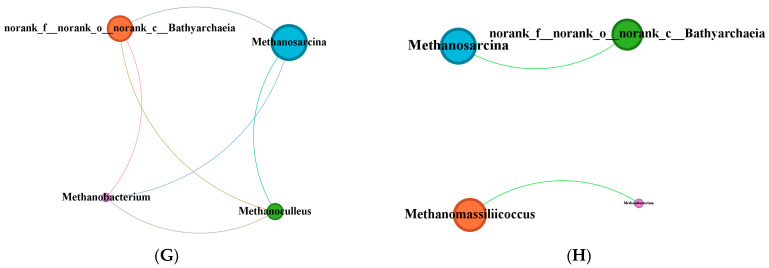
Co-occurrence networks of microbial genera in the fermentation broth after co-degradation with different coal-to-sawdust ratios. Each node represents a microbial genus, with node size proportional to microbial abundance. Node color indicates the functional group to which the microbe belongs. Connections between nodes signify statistically significant co-occurrence relationships, where red denotes positive correlation and green denotes negative correlation. Bacterial co-occurrence networks for each experimental group are shown in: (**A**) KB, (**B**) MM, (**C**) MT, and (**D**) MX. Archaeal co-occurrence networks for each experimental group are shown in: (**E**) KB, (**F**) MM, (**G**) MT, and (**H**) MX. KB, pure coal group; MM, coal-to-sawdust ratio of 4:1; MT, coal-to-sawdust ratio of 1:1; MX, pure sawdust group.

To further investigate the role of the core microorganisms in the microbial community, co-occurrence networks were constructed based on genus-level correlations (Spearman’s correlation > 0.7, *p* < 0.01) for the KB, MM, MT, and MX groups (Figure 7). Analysis of bacterial communities revealed distinct topological features (Table 4). The number of nodes and edges in the MX group was markedly lower than that in the coal-containing group. The modularity index of the bacteria in all four experimental groups was greater than 0.4, whereas the average clustering coefficient and average path length values were consistently 1 across the groups. A detailed comparison revealed the following trends: average degree, KB > MT > MM > MX; graph density, MX > KB > MT > MM; and average eigenvector centrality, MM > MT > MX > KB. Analysis of archaeal communities indicated relatively simple network structures in all groups, with fewer nodes and edges (Table 5). The average degree followed the order of MM > MT > KB > MX, graph density values were ranked as MM = MT > KB > MX, and average eigenvector centrality values were ranked as MM > MT = MX > KB (Table 6).

## 4. Discussion

From the perspective of coal stoichiometry, the lack of hydrogen element limits the formation of biogenic coalbed methane. Biomass is rich in hydrogen element. Guo et al. [26] found that the methane yield from co-fermentation of rice straw, sweet sorghum straw, wheat straw, and corn straw with coal was higher than that from fermentation of any single material. By comparing hydrogen production in co-fermentation and single fermentation, it was suggested that the increase in methane production may be attributed to enhanced biodegradation of coal rather than the supply of additional hydrogen. Guo et al. [27] investigated the effects of different types of waste kitchen oil on biomethane production from lignite. Compared with anaerobic fermentation using lignite alone, the cumulative methane production from mixed anaerobic fermentation of waste kitchen oil and lignite increased by 377.86%. This was attributed to the fact that waste kitchen oil increased the content of volatile fatty acids associated with biomethane production. In another study, Guo et al. [28] investigated how coal slime as an additive influences methane production. The aim was to improve methane yield from anaerobic digestion of chicken manure. The results showed that adding a suitable amount of coal slime increased the gene abundance of key enzymes involved in methane metabolism. This enhanced the initiation of fermentation. Based on the above studies, it has been demonstrated that co-fermentation of coal with other organic materials can enhance methane production. However, systematic studies on how organic matter enhances methane production from coal conversion remain limited.

This study investigated the effectiveness of co-fermentation of coal with sawdust. Compared to degradation of coal alone, the mixed degradation of coal and sawdust exhibited higher efficiency. Previous studies have indicated that co-digestion of lignocellulose-rich components (such as straw) with coal can increase the H/C ratio in the degradation substrate [3,29], thereby promoting biomethane production. However, in this study, methane production did not show a simple linear relationship with the amount of sawdust added, which is speculated to be related to the composition of the degradation matrix. The generally accepted optimal C/N ratio for anaerobic fermentation ranges from 20 to 30 [30,31]. Within this range, sufficient nitrogen source enables microorganisms to synthesize adequate enzymes and proteins, supporting their growth and metabolic activities and ensuring effective system degradation [32,33]. However, the addition of sawdust increased the C/N ratio beyond this range, which may lead to the accumulation of VFA, subsequently causing acidification [34], impairing microbial cell function and unbalancing the anaerobic fermentation system [35]. Surprisingly, in this study, compared with coal-alone degradation, the 4:1 group showed a significant increase in methane yield, up to 7.97 times higher (Figure 1A). This may be because sawdust are easily degraded by microorganisms, providing immediate energy and carbon sources, which stimulated microbial activity and enabled timely degradation of the accumulated organic acids—resulting from the increased C/N ratio—into methane, thereby reducing acid inhibition. Nevertheless, although sawdust have a high H/C ratio, their individual degradation yielded very little methane, which may be related to the structure, polymerization degree, surface functional groups, pore volume, and specific surface area of lignocellulose [36,37].

Untargeted metabolomics was employed to investigate the changes in metabolites during coal degradation. The fermentation broth contained substantial amounts of soluble organic carbon, and the introduction of sawdust promoted the degradation of organic matter in the coal. This altered the dissolved organic matter in the fermentation broth (Figure 2). Among these metabolites, alkanes, long-chain fatty acids, phenols, and low-molecular-weight aromatic hydrocarbons are important reactant [38,39]. This study focused on quantitative analysis of the characteristic organic compounds during the reaction. When sawdust was added, alcohols, phenols, aldehydes, and ketones exhibited systematic variations. During co-fermentation, the organic compounds in the broth became less diverse and structurally simpler, while the proportion of hydrocarbons increased. This implied that the addition of sawdust facilitated the conversion of organic matter into methane precursors. Under anaerobic conditions, microorganisms break down aromatic rings via reductase and other electron acceptors to obtain energy from carbon. [36]. However, as the coal rank increases, the proportion of conjugated aromatic rings per structural unit in the coal matrix increases, leading to a decreased degradation rate of polycyclic aromatic hydrocarbons [40,41]. Lignin-derived aromatic compounds serve as carbon and energy sources for microorganisms. Furthermore, they may enhance the efficiency of converting lignin into value-added chemical products. During this process, benzene rings are cleaved to form monoaromatic and aliphatic compounds [12,42], which is consistent with the findings of this study. Elevated levels of ethylbenzene and xylene were detected in the mixed groups. This result indicates that adding sawdust accelerated the ring-opening and cleavage processes of aromatic hydrocarbons. Furthermore, fatty acids, lipids, and organic acids, which are key products of microbial fermentation and metabolic activity [43,44], were found at lower concentrations in the mixed groups than in the control groups. This reduction was most pronounced at a coal-to-sawdust ratio of 4:1. The observed differences in key enzyme activities likely resulted from the varying substrates. This difference enabled the mixed groups to utilize more target products via synergistic metabolism. In contrast, the experimental group accumulated metabolites due to a more restricted metabolic pathway.

In this study, the original sawdust and the solid products from the MX group served as the control. These were compared against the solid products from the experimental groups (MT and MM). The comparison revealed a significant decrease in the relative lignocellulose content after the reaction. Cellulose, hemicellulose, and lignin in sawdust are insoluble in water, diluted acids, and alkaline solutions at room temperature [45]. This confirms that the consumption of lignocellulose in this experiment was related to anaerobic degradation (Figure 3A). Sawdust addition introduced readily degradable organic matter. This organic matter stimulated the proliferation of functional microorganisms. These microorganisms are capable of producing cellulases and hemicellulases. To some extent, this alleviated the potential inhibitory effect of coal on microorganisms and created a suitable microenvironment for hydrolytic bacteria, such as *Lentimicrobium* and *Desulfovibrio*. After anaerobic degradation, the calorific value of the solid residues decreased to varying degrees compared with that of the original samples (Figure 3B). This is due to the conversion of chemical energy in the degradation substrate via microbial metabolism into biological energy, chemical energy in gaseous products, and heat energy lost during energy conversion. Thus, the ratio of the calorific value of the degraded substrate to that of the products reflects the efficiency of microbial substrate utilization during degradation. The experimental data indicate that the addition of sawdust improves the conversion rate of the degradation substrate.

To some extent, changes in the microbial community structure corresponded with variations in the organic components in the fermentation broth; however, the response patterns of richness and diversity differed. In this study, the α-diversity indices of archaeal communities did not differ substantially among the experimental groups (Table 5). This result indicated that coal-sawdust co-fermentation did not significantly alter archaeal species richness or community complexity. This stability suggests strong metabolic specialization and niche conservatism in the archaeal community. The addition of sawdust promoted the metabolism of certain functional bacteria, leading to increased bacterial richness and diversity in the MM group. However, as the woodchip content increased, the differences in community parameters between the MT and MX groups and the KB group decreased (Table 4). This may be because excessively high proportions of sawdust caused the fermentation system to shift toward a “single-carbon source degradation” state. This resulted in functional redundancy, intensified intercommunity competition, a higher proportion of dominant species, and gradual simplification of the fermentation system, which was similar to the observations during pure coal degradation. This demonstrates that bacterial communities respond more sensitively to changes in the fermentation environment.

Changes in the substrate altered the niche partitioning of the bacterial taxa (Figure 5). In the KB group, the bacterial community was complex, evenly distributed, and lacked dominant species, which could be attributed to the recalcitrant nature of coal [46]. In contrast, as the proportion of sawdust in the substrate increased, functional microorganisms, particularly *Bacillus* and *Clostridium*, and thermophilic cellulose-degrading bacteria gradually became dominant in the fermentation system. These genera are closely associated with the formation of hydrogen and acetate [47]. An increase in dominant genera improved the degradation efficiency of aromatic hydrocarbons and cellulose. Furthermore, *Desulfovibrio*, which was present in the fermentation broth, often forms syntrophic relationships with methanogens. They oxidize carbohydrates and small-molecule organic acids via carboxylate transporter systems. This activity plays a key role in coal organic matter degradation and biomethane production [48,49].

The KB group exhibited distinct differences from the other groups, whereas the addition of sawdust markedly weakened this specific selection pressure on the core archaeal taxa (Figure 6), leading to more consistent community structures across the co-fermentation groups. This suggests that coal exerts a stronger selective pressure on archaea, whereas sawdust act as a buffer that mitigates this pressure and promotes niche release for certain taxa. The inclusion of sawdust increased the relative abundance of unclassified genera in the Bathyarchaeia. This group is capable of homoacetogenesis in the absence of methyl-coenzyme M reductase and links sulfur redox reactions to fermentation processes, thus indicating a potential syntrophic relationship with methylotrophic methanogens [50,51]. This aligns with the observed abundance of *Desulfovibrio* and *Methanosarcina* in this study. The relative abundance of *Methanosarcina* remained consistent across groups, which may be attributed to its high metabolic versatility [52]. Other archaea, such as the hydrogenotrophic *Methanoculleus* and methylotrophic *Methanomassiliicoccus* [53], exhibited distinct distribution patterns. This indicates that varying coal-to-sawdust ratios may selectively enrich specific functional archaeal genera by altering substrate degradability.

The biological communities within the reactors exhibited clear niche-specific characteristics (Figure 7). Analysis of the bacterial co-occurrence network revealed a compact “small-world” architecture with high microbial activity and strong interactions. A high average degree indicates strong co-occurrence relationships among microbial taxa during anaerobic degradation. As the proportion of sawdust in the substrate decreased, the community integration increased, with more complex and frequent species interactions. This likely stems from the recalcitrance and structural complexity of coal, which necessitates more intricate degradation pathways than those of sawdust. A high average eigenvector centrality indicates the presence of influential “core” species. These species form a hierarchical structure, which corresponds to the multi-stage reactions in anaerobic degradation. The bacterial communities in the experimental groups were more dependent than those in the control group on core species. This may imply reduced robustness but could also enable more efficient signal transduction. High graph density indicates tight internal connections and strong species interdependence. However, the highly integrated bacterial communities did not increase methane production. This may be attributed to the highly heterogeneous structure of coal, the non-stoichiometric nature of coal degradation reactions, and enzyme non-specificity. Although coal provides diverse degradation pathways, the concentration of bioavailable organic compounds in the liquid environment remains low. This limitation amplifies both functional redundancy and resource competition among microorganisms. Consequently, the overall efficiency of the system is reduced. In contrast, sawdust have a simpler structure and are more easily degraded, thereby releasing nutrients into the liquid environment. This explains why the graph density in the coal-containing groups was inversely correlated with sawdust content. Owing to substrate homogeneity, the MX group exhibited simpler degradation pathways, tighter bacterial connections, and higher graph densities than those of the coal-containing groups. Analysis of the archaeal communities indicated low diversity and high cohesion, with strong interactions among the archaeal genera. The addition of sawdust stimulated the emergence of functional archaea. This enhanced network complexity and stability while altering the dominant archaeal groups in the fermentation broth. Consequently, methanogenic pathways within the system were modified. These findings provide ecological insights into the assembly and functional dynamics of microbial communities in extreme environments.

This study investigated the enhancement of microbial methane production from low-rank coal through anaerobic co-degradation with sawdust. First, the experiments were carried out in small-scale batch reactors, so it remains uncertain whether the results would apply to continuous or large-scale operations. Second, the reliable supply of sawdust, its cost-effectiveness, and practical compatibility in real industrial settings need further assessment. Additionally, potential environmental effects—such as by-products and wastewater generated during co-degradation—are still not well understood, highlighting the need for broader sustainability analysis in future studies. Future work should focus on pilot-scale validation, optimization of substrate matching, and comprehensive energy-environment benefit evaluations to facilitate the transition of this technology toward industrial application.

## 5. Conclusions

This study confirms that adding sawdust significantly enhances the microbial gasification efficiency of coal. At a coal-to-wood-chip ratio of 4:1, methane production reached its peak (49.28 mL), representing an approximately 7.96-fold increase compared to the pure coal group (6.19 mL). The inclusion of sawdust accelerated the degradation of aromatic hydrocarbons and lipid compounds within the substrate, while improving microbial community structure. This led to a significant increase in the abundance of functional bacteria such as *Bacillus* and *Clostridium*, enhanced species interactions, and a more defined metabolic division of labor. Additionally, sawdust mitigated the environmental stress imposed by coal on microorganisms, thereby improving organic matter utilization efficiency. In summary, wood chip co-degradation represents an effective intensification strategy for promoting biological methane production from low-rank coal.

## Figures and Tables

**Table 1:** Industrial analysis of lignite.

	M (%)	Ad (%)	Vd (%)	Fcd (%)	V (%)
Lignite	16.43	9.59	35.59	38.39	47.96

**Table 2:** Industrial analysis of sawdust.

	M (%)	Ad (%)	Vd (%)	Fcd (%)	V (%)
Sawdust	28.86	0.21	62.28	8.65	/

**Table 3:** Organic composition in fermentation broth.

Number	Compound	Time (min)	Molecular Formula	MX	MT	MM	KB
				Present (%)			
1	Ethylbenzene	5.123	C_8_H_10_	3.19	3.52	10.46	10.14
2	Benzene, 1,3-dimethyl-	5.256	C_8_H_10_	19.76	21.99	63.66	66.41
3	Heptane, 3,3,5-trimethyl-	7.847	C_10_H_22_	0.91	/	3.04	2.58
4	Dodecane, 4,6-dimethyl-	8.420	C_14_H_30_	1.46	/	5.23	3.78
5	Nonane, 5-methyl-5-propyl-	8.422	C_13_H_28_	0.55	1.09		/
6	Heneicosane	11.537	C_21_H_44_	0.55	0.80		2.32
7	Hexadecyl iodide	11.765	C_16_H_33_I	0.99	/		/
8	Heptadecane, 8-methyl-	11.765	C_18_H_38_			3.37	/
9	Dotriacontane	14.592	C_32_H_66_	/	/	3.20	/
10	4,4,7-Trimethyl-oct-5-enal	16.709	C_11_H_20_O	4.18	/	/	/
11	Longifolene	17.286	C_15_H_24_	4.14	/	/	/
12	4,8,13-Cyclotetradecatriene-1,3-diol, 1,5,9-trimethyl-12-(1-methylethyl)-	17.360	C_20_H_34_O_2_	5.88	/	/	/
13	2-Naphthalenemethanol, 2,3,4,4a,5,6,7,8-octahydro-α,α,4a,8-tetramethyl-, [2R-(2α,4aβ,8β)]-	18.369	C_15_H_26_O	11.75	/	/	/
14	Carotol	18.483	C_12_H_20_O	6.11	/	/	/
15	1H-Inden-1-ol, 2,4,5,6,7,7a-hexahydro-4,4,7a-trimethyl-	18.871	C_12_H_20_O	6.59	/	/	/
16	2-tert-Butyl-6-(3-tert-butyl-2-methoxy-5-methylbenzyl)-4-methylphenol	19.059	C_24_H_34_O_2_	3.63	/	/	/
17	3-n-Heptyl-7-methyl-9-(2,6,6-trimethylcyclohex-1-enyl)nona-2,4,6,8-tetraenal	19.144	C_26_H_40_O	5.21	/	/	/
18	Dibutyl phthalate	19.761	C_16_H_22_O_4_	9.98	3.99	11.05	10.74
19	Cyclic octaatomic sulfur	20.703	S_8_	10.25		/	/
20	4-Camphenylbutan-2-one	21.899	C_14_H_22_O	4.89		/	/
21	1,5-Diphenylbicyclo[3.2.0]heptane	24.818	C_19_H_20_		2.29	/	/
22	Benzene, 1,1′-[3-(2-phenylethylidene)-1,5-pentanediyl]bis-	26.070	C_25_H_26_	/	29.46	/	/
23	13-Docosenamide, (Z)-	26.360	C_22_H_43_NO	/	10.74	/	4.04
24	(1-Benzyl-2-O-tolyl-ethyl)-isonitrile	26.995	C_17_H_17_N	/	13.35	/	/
25	Tricyclo[6.6.0.0(3,6)]tetradeca-1(8),4,11-triene	27.706	C_14_H_18_	/	2.90	/	/
26	3-(2-Benzyl-benzoimidazol-1-yl)-propane-1,2-diol	27.854	C_17_H_18_N_2_O_2_	/	3.88	/	/
27	1,5-Hexadiene, 2,5-bis(4-methylphenyl)-	28.05	C_20_H_22_	/	5.99	/	/

**Table 4:** Alpha diversity indices of bacterial communities.

Sample\Estimators	Ace	Chao	Coverage	Shannon	Simpson	Sobs
KB_1	268.2323	278.4545	0.998783	3.133265	0.104061	225
KB_2	232.9451	228.5652	0.999056	3.437144	0.050625	198
KB_3	241.823	241.6154	0.999081	3.243105	0.08023	216
MM_1	292.6472	285.0233	0.998882	1.99676	0.359385	262
MM_2	313.3657	315.8333	0.998758	2.712693	0.187143	275
MM_3	318.2038	311.4667	0.998708	2.127637	0.280326	282
MT_1	279.2989	278.5517	0.998783	2.443099	0.223104	238
MT_2	275.9088	269.5882	0.998783	2.900184	0.124126	235
MT_3	286.1446	272.0222	0.998907	2.847191	0.134001	251
MX_1	328.2939	319.1277	0.998907	2.950831	0.143841	299
MX_2	347.1461	339.5	0.998783	2.982618	0.140831	315
MX_3	312.4448	311.3871	0.998808	2.840316	0.164025	275

KB, pure coal group; MM, coal-to-sawdust ratio of 4:1; MT, coal-to-sawdust ratio of 1:1; MX, pure sawdust group.

**Table 5:** Alpha diversity indices of archaea communities.

Sample\Estimators	Ace	Chao	Coverage	Shannon	Simpson	Sobs
KB_1	19.8151	14.5	0.999898	1.443567	0.280196	13
KB_2	13	12	0.999966	1.468794	0.263044	12
KB_3	24.13108	20	0.999898	1.010594	0.531176	19
MM_1	14.45361	14	0.999966	1.102896	0.388644	14
MM_2	12.54519	12	0.999966	0.998595	0.439577	12
MM_3	12.69444	12	0.999966	1.049663	0.410967	12
MT_1	17.48	17	0.999932	1.428108	0.283833	16
MT_2	16.92593	15.33333	0.999932	1.354257	0.309725	15
MT_3	18.15107	15.5	0.999932	1.297845	0.336386	15
MX_1	13	13	1	1.095285	0.437145	13
MX_2	13.62432	13	0.999966	1.232242	0.35422	13
MX_3	12	12	1	1.378394	0.273715	12

KB, pure coal group; MM, coal-to-sawdust ratio of 4:1; MT, coal-to-sawdust ratio of 1:1; MX, pure sawdust group.

**Table 6:** Parameters of Collinearity Networks.

Sample	Node	Edge	Average Degree	Graph Density	Modularity	Avg. Clustering Coefficient	Avg. Path Length	Avg. Eigenvector Centrality
a	105	1507	29.1	0.282	0.576	1	1	0.517
b	100	1325	25.4	0.245	0.671	1	1	0.658
c	99	1342	26.2	0.257	0.656	1	1	0.657
d	70	819	22.2	0.304	0.605	1	1	0.543
e	5	4	1.6	0.4	0.375	1	1	0.030
f	5	10	4	1	0	1	1	0.050
g	4	6	3	1	0	1	1	0.040
h	4	2	1	0.333	0.5	1	1	0.040

## Data Availability

Data will be made available on request.

## Abbreviations

The following abbreviations are used in this manuscript:

GC-MS	Gas Chromatography–Mass Spectrometry
MANOVA	Multivariate analysis of variance
PCR	polymerase chain reaction
OTU	operational taxonomic unit
MM	coal-to-sawdust ratio of 4:1
MT	coal-to-sawdust ratio of 1:1
KB	pure coal group
MX	pure sawdust group
